# An industry perspective on the use of machine learning in drug and vaccine safety

**DOI:** 10.3389/fdsfr.2023.1110498

**Published:** 2023-02-01

**Authors:** Jeffery L. Painter, Raymond Kassekert, Andrew Bate

**Affiliations:** ^1^ GlaxoSmithKline, Global Safety, Durham, NC, United States; ^2^ GlaxoSmithKline, Global Safety, Upper Providence, PA, United States; ^3^ GlaxoSmithKline, Global Safety, Brentford, Middlesex, United Kingdom; ^4^ London School of Hygiene and Tropical Medicine, London, United Kingdom

**Keywords:** pharmacovigilance, machine learning-ML, drug safety, vaccines safety, artificial intelligence

## Abstract

In recent years there has been growing interest in the use of machine learning across the pharmacovigilance lifecycle to enhance safety monitoring of drugs and vaccines. Here we describe the scope of industry-based research into the use of machine learning for safety purposes. We conducted an examination of the findings from a previously published systematic review; 393 papers sourced from a literature search from 2000–2021 were analyzed and attributed to either industry, academia, or regulatory authorities. Overall, 33 papers verified to be industry contributions were then assigned to one of six categories representing the most frequent PV functions (data ingestion, disease-specific studies, literature review, real world data, signal detection, and social media). RWD and social media comprised 63% (21/33) of the papers, signal detection and data ingestion comprised 18% (6/33) of the papers, while disease-specific studies and literature reviews represented 12% (4/33) and 6% (2/33) of the papers, respectively. Herein we describe the trends and opportunities observed in industry application of machine learning in pharmacovigilance, along with discussing the potential barriers. We conclude that although progress to date has been uneven, industry is very interested in applying machine learning to the pharmacovigilance lifecycle, which it is hoped may ultimately enhance patient safety.

## 1 Introduction

The vast increase in the volume of safety reporting over the last years was only exacerbated during the global COVID-19 pandemic. The introduction of new vaccines and medicines in response to the pandemic resulted in more than 1.8 million new safety reports. Enabling the safety community to cope with the onslaught of data has led to increased interest in automating pharmacovigilance (PV) activities within the pharmaceutical industry and prompted safety organizations to advance their technological capabilities (Rudolph et al., 2022).

**FIGURE 1:**
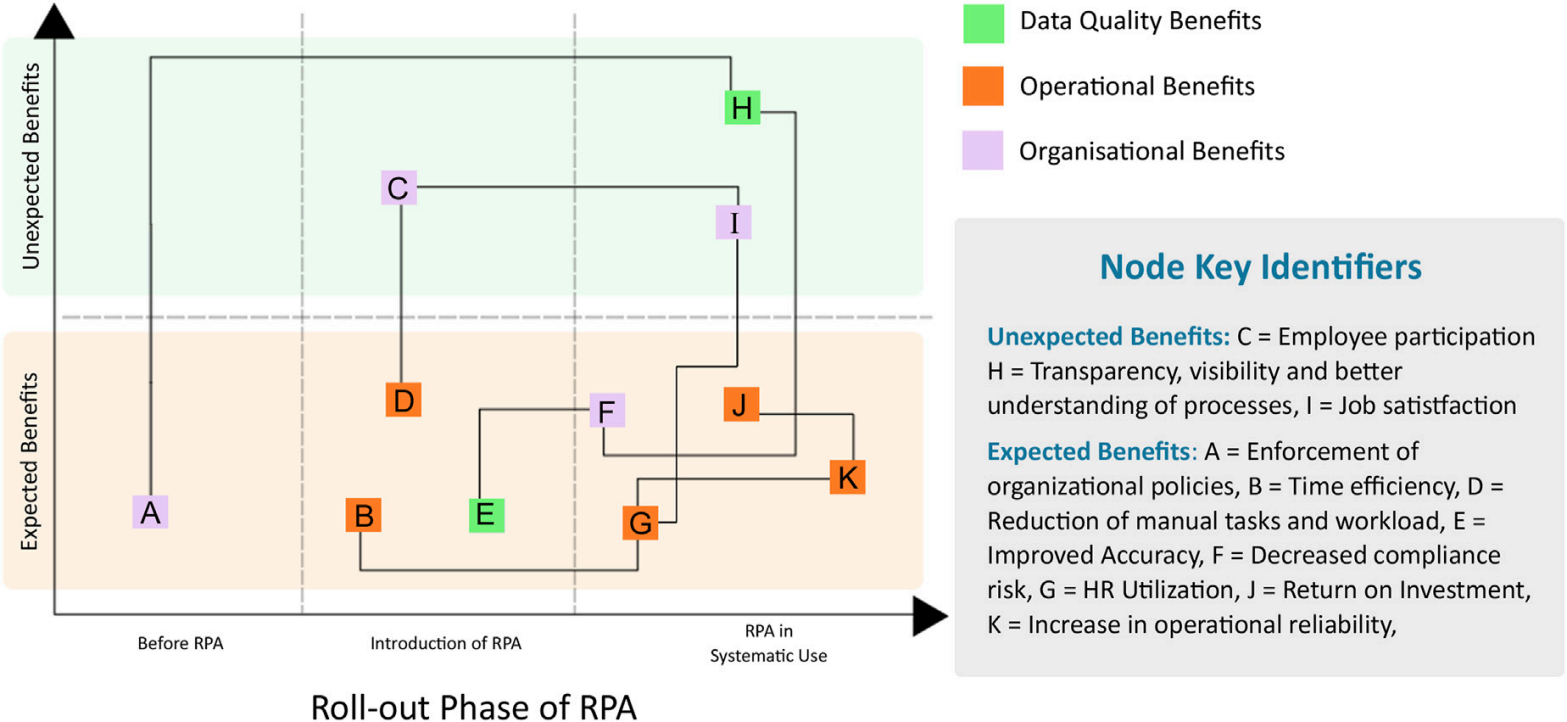
The benefits of robotic process automation.

Even before the pandemic, automation and its potential benefits for PV activities were well recognized (Kassekert et al., 2022). Rules based automation, also known as robotic process automation (RPA), are well established and have been routinely used for several years by companies to assist in the processing of individual case safety reports [ICSRs; see (Kassekert et al., 2020) for an example]. Here, we have adapted the RPA benefit diagram first described by (Vitharanage et al., 2020). Benefits realized through RPA include increased timeliness of data ingestion, higher quality case processing, and reduction in the manual efforts required of case processors. Furthermore, through automation, additional, indirect benefits to PV functions have been realized, such as increased operational reliability, improved job satisfaction, and increased transparency in data management processes (Figure 1). Thematic groups of the nodes are identified by their color, while interdependencies between graph nodes are deduced intuitively and deserve future investigation to refine. Each node of the RPA graph is expanded in greater detail with full descriptions in (Table 1).

**TABLE 1:** Details on the benefits of robotic process automation (RPA).

Node	Phase	Benefit	Description
Expected Benefits			
A	Before RPA	Enforcement of organizational policies	Automation defaults to observing organizational policies in a systematic way
B	Introduction of RPA	Improvement in time efficiency	Reduction in time for case processing and booking
D	Introduction of RPA	Reduction of manual tasks and workload	Reduction of repetitive, mundane, tedious manual tasks
E	Introduction of RPA	Improvement in data accuracy	Automate data processing in systematic way. Decrease potential errors and mistakes in safety case review and processing
F	RPA in Systematic Use	Decrease in compliance risk	Timelier fulfillment of compliance and audit requirements for regulatory bodies
G	RPA in Systematic Use	Improvement in human resource utilization	Better utilization of staff, focus on cases which require human intervention
J	RPA in Systematic Use	Return on investment	Increase capability for high volume case processing without increasing staff
K	RPA in Systematic Use	Increase in operational reliability	Allows the organization to operate reliably, even when faced with unexpected challenges
Unexpected Benefits			
C	Introduction of RPA	Improved employee participation	Staff is relieved from repetitive tasks and able to focus on improving overall business processes and decision making
H	RPA in Systematic Use	Improvement in transparency, visibility and better understanding of processes	Business processes and business rules are judiciously executed, processes are documented and explainable to stakeholders
I	RPA in Systematic Use	Improvement in job satisfaction	Empower staff to focus on the most important tasks which lead to better job fulfillment

There has been a growing interest in applying machine learning (ML) across the entire PV lifecycle, from data ingestion and quality control to regulatory or health authority reporting and signal detection. Currently, it is not obvious to what extent ML can be used for routine, operational PV activities and the practical implications this will have for patient safety. More simplistic ML applications such as association rule analysis, or disproportionality analysis, have been used routinely for signal detection in most organizations (Bate and Evans, 2009). Spontaneous safety reports are routinely analyzed using this family of methods as part of holistic PV management systems (Almenoff et al., 2007). The use of ML in other PV applications however is not routine, although there are isolated reports of ML use for purposes other than signal detection, for example, detection of duplicate spontaneous reports (Norén et al., 2007; Bate and Luo, 2022).

The objective of this paper is to describe the scope of industry-based research into the use of ML for safety purposes, and in particular PV.

## 2 Unravelling the routine use of ML in PV

A recently published systematic review on the history and use of ML in PV was conducted by evaluating the published literature from 2000–2021 (Kompa et al., 2022). In this scoping review, 393 papers met the criteria for analysis and were analyzed across several metrics including types of safety data used, types of ML models built, and specific PV subtasks addressed. We conducted a thorough examination of the findings of the systematic review (Kompa et al., 2022) to derive a better understanding of the routine use of ML by the pharmaceutical industry and herein present our findings. First, the contributors and affiliations of the 393 papers were reviewed and each paper was attributed to one of three sources (industry, academia, or regulatory authorities). To be included in the original systematic review, the published literature was limited to English papers including “ML terms related to disproportionality analysis, common to PV research, as well as modern ML techniques (e.g., deep learning).” (Kompa et al., 2022). This analysis is subject to these same limitations, and possibly excludes active ML work in the industry which has not been published in peer reviewed literature.

### 2.1 Attribution assignment

We created an algorithm to automatically perform the attribution function, by searching the authors and affiliations, conflict of interest statements, and sources of funding (grants or sponsorship) of the 393 papers. A list of the top 80 pharmaceutical companies was compiled to allow us to link a particular paper to one or more companies through keyword identification. Regulatory papers were identified similarly using a smaller subset of terms (e.g., FDA, EMA) while the remaining articles were labeled academic papers which did not contain either an industry partner name or regulatory affiliation in either the articles authorship or funding disclosures. The algorithmic generated results for industry-associated publications were then manually reviewed and verified.

Initially, industry-associated articles in this analysis include papers that were either sponsored by one of the companies on the list or have at least one author affiliated with one of the companies on the list. However, in practice, for these industry-associated publications, a broad inclusive definition of ‘industry’ was taken based on text written in the publications. Publications were included where pharmaceutical industry were involved directly, as co-authors or according to acknowledgements, or sources of funding (i.e., had funded or partially funded the works). Additionally, if more than one academic author declared industry funding, even if not directly related to the manuscript, we considered it in scope as the foundational work funded by industry could have influenced the work presented in the article. Moreover, as the list of publications was generated for this manuscript using an automated objective filter of the original systematic review, manual review of the output was then used to remove articles not fulfilling our definition of ‘industry’ for this manuscript from the analysis set.

Forty-three papers were attributed to industry by the algorithmic process, and after manual review of these papers, 33 were verified to be industry contributions. Most of the misclassifications were due to partial term matches of company names. There were less than five articles where the paper did include a company author, but the work was largely regulatory sponsored, and other misses included product mentions of a pharmaceutical company that was not leading the investigation of the manuscript in question. For each of the classifications, all papers were assigned to only one of the three categories (industry, academic, or regulatory).

**FIGURE 2:**
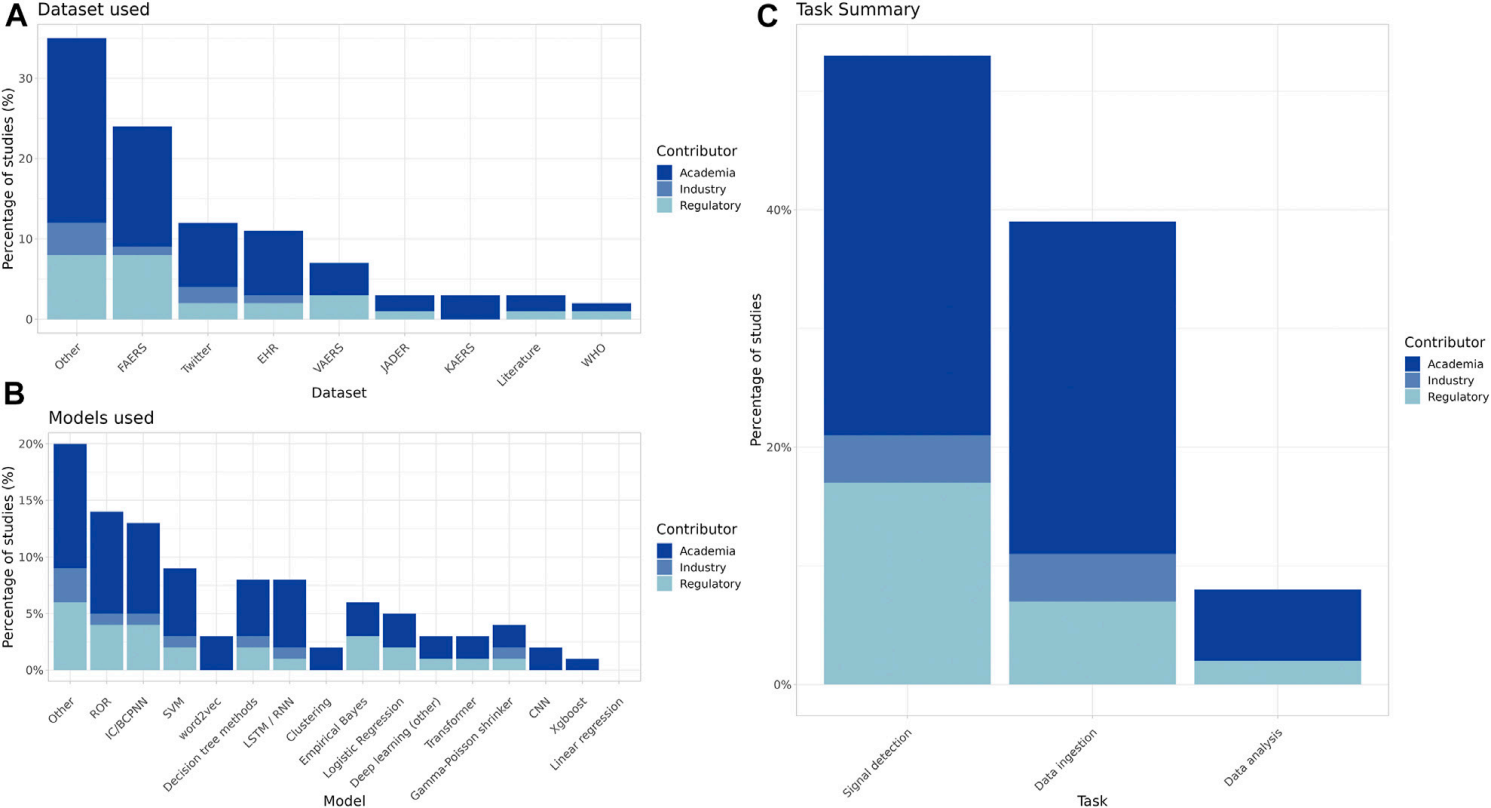
Summary of datasets used, primary algorithms, and task type of the included studies by contributor (**(A)**: FAERS = FDA Adverse Event Reporting System, EHR = Electronic Health Records, VAERS = Vaccine Adverse Event Reporting System, JADER = Japanese Adverse Event Reporting System, KAERS = Korean Adverse Event Reporting System, WHO = World Health Organization VigiBase **(B)**: ROR = Reporting Odds Ratio, IC/BCPNN = Information Component/Bayesian Confidence Propagation Neural Network, SVM = Support Vector Machine, LSTM/RNN = Long short-term memory/Recurrent Neural Network, CNN = Convolutional Neural Network). **(C)** The most common PV tasks for processing and evaluating safety reports include at a high level data ingestion, data analysis and signal detection.

The breakdown by contributor type provides insight as to the datasets used, the types of models constructed, and the specific PV tasks being addressed by industry, academia, and regulatory bodies (Figure 2A–C).

**FIGURE 3:**
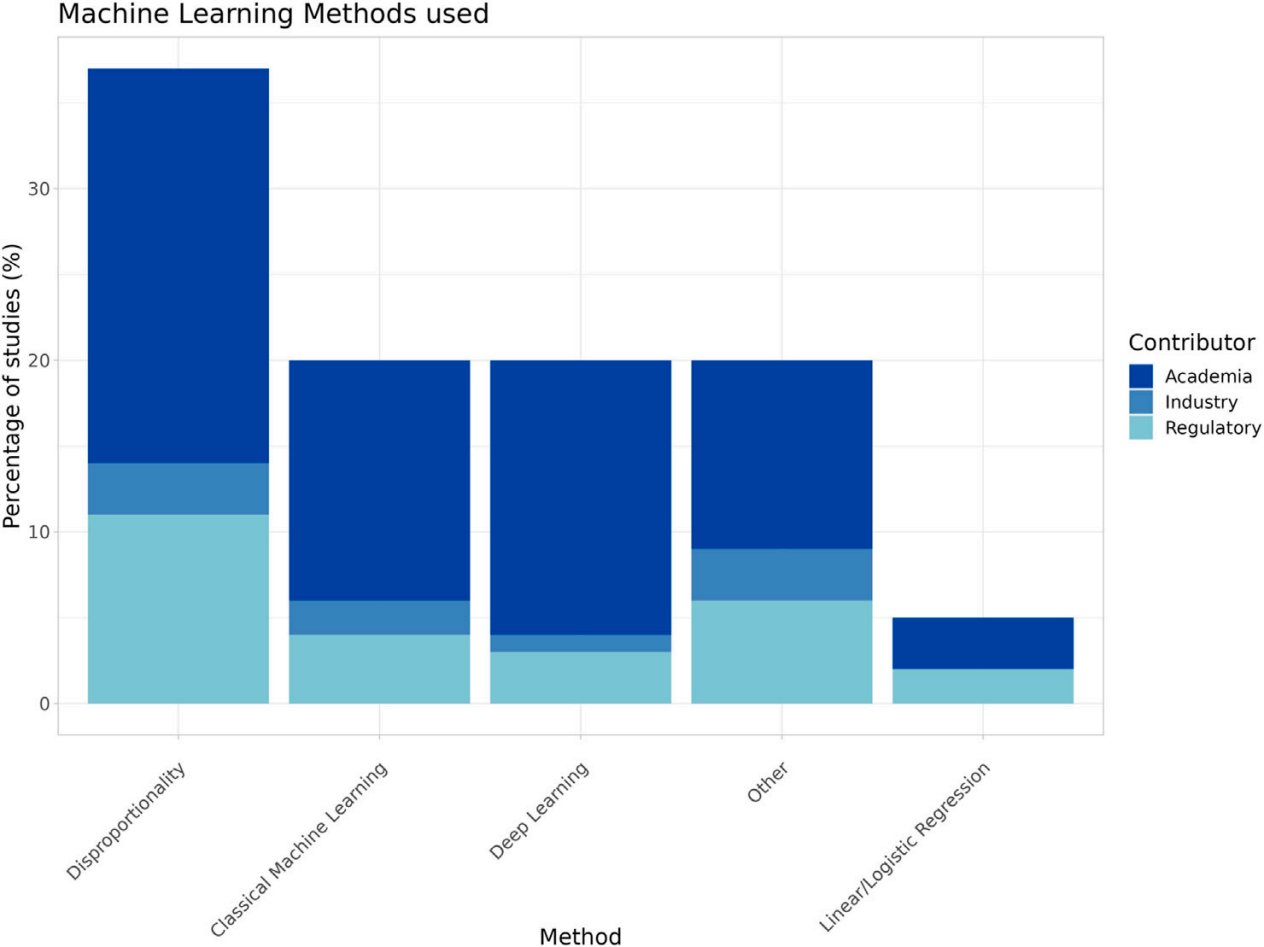
Machine learning methods employed by contributor.

Many papers used traditional disproportionality analysis (DPA) methods (Figure 3), and such studies made up most of the signal detection research papers across all contributor types.

### 2.2 Industry trend analysis

**FIGURE 4:**
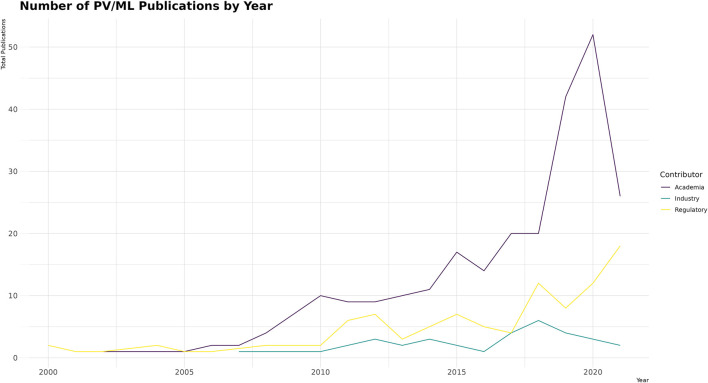
Number of PV/ML applications published yearly by contributor.

When looking at the total number of publications devoted to ML in PV by year (Figure 4), academia dominates, followed by regulatory bodies (e.g., the US Food and Drug Administration, World Health Organization, and Centers for Disease Control and Prevention), whereas industry makes up a small percentage of the overall publications in the field (8.4%, 33/393).

Over the period analyzed (2000–2021), the number of papers attributed to industry, academia, and regulatory authorities has generally increased (Figure 4). Industry appears to have lagged behind academia in the early 2000s, although some articles that could be attributed to industry were not included in the review (Fram et al., 2003; DuMouchel et al., 2004), and it must be acknowledged that some work in ML originating from industry predates the time period included of the review (Alvager et al., 1994; Bate et al., 1998). It may be speculated that industry is not prioritizing publishing these types of papers in the way academia is, and therefore industry may be underrepresented.

Of the 33 industry-associated papers included in the analysis, 19 58% (19/33 = 57.6) were attributed to one of nine different companies, and 14 (42%) were collaborative works that included authors from more than one company.

### 2.3 Major trends and opportunities in industry application of ML in PV

**FIGURE 5:**
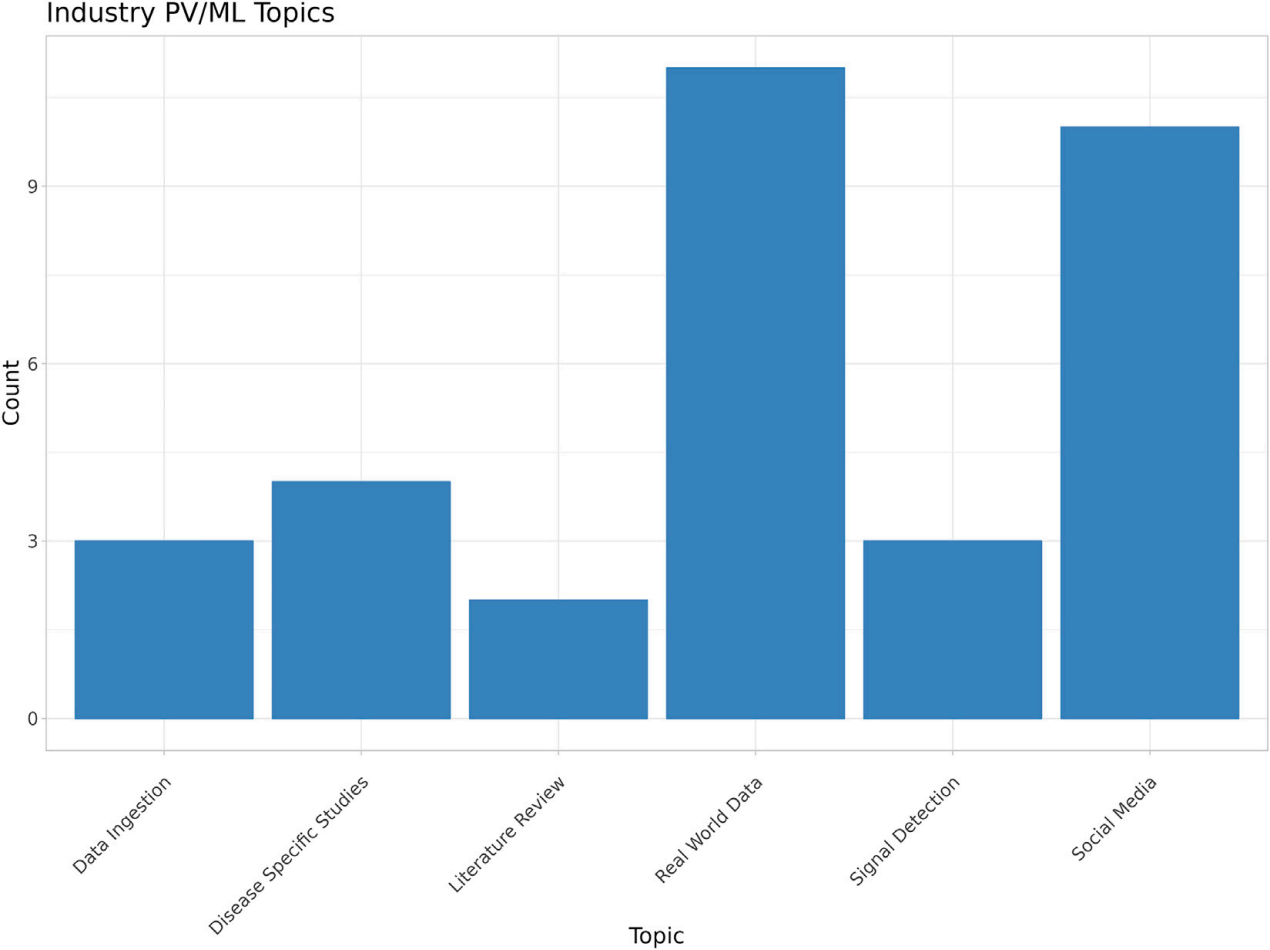
Major trends and topics relating to machine learning in industry-attributed papers (Classical machine learning includes typical statistically based ML methods such as NLP, clustering and classification tasks not based on neural networks).

During the manual review process, each of the 33 industry-associated papers was assigned to one of six primary categories. These were defined by the authors to capture the major PV functions performed by ML in each study. These categories included data ingestion, disease-specific studies, literature review (i.e., for signal detection), leveraging real world data (RWD), signal detection in spontaneous safety reports, and the use of social media data (Figure 5). Topic assignments to each paper were performed manually and can be found in the supplementary data.

RWD (Nordstrom et al., 2007; Gurulingappa et al., 2012a; Cao et al., 2013; Cheetham et al., 2014; Ferrajolo et al., 2014; Yeleswarapu et al., 2014; Whalen et al., 2018; Chapman et al., 2019; Choudhury et al., 2019; Wintzell et al., 2020; Fralick et al., 2021) and social media (Jimeno-Yepes et al., 2015; Powell et al., 2016; Cocos et al., 2017; Curtis et al., 2017; Pierce et al., 2017; Comfort et al., 2018; Gupta et al., 2018; Masino et al., 2018; Gavrielov-Yusim et al., 2019; Gartland et al., 2021) were the most frequent PV functions represented and, collectively, comprised 63% (21/33) of the papers included in the review. This is not surprising, as the use of social media to supplement PV activities began around 2009, with broader use of social media by the public, and attracted interest from both industry and academia as a potential source of safety-related events in near real time. However, more recently, interest in social media data use has declined because of accumulating evidence of variable data quality which limits its value for much (but not all) PV (van Stekelenborg et al., 2019; Powell et al., 2022). In addition, most social media data is typically characterized as unstructured data requiring ML-based methods in order to attempt to glean any insights related to PV activities (Comfort et al., 2018).

RWD has long been used by industry for pharmacoepidemiologic studies and across the drug and vaccine development lifecycle (Bate et al., 2016; Gatto et al., 2019; Garcia-Gancedo and Bate, 2022). The use of ML for the wider use of RWD continues to attract interest for routine PV activities, and we anticipate this trend to continue. Like social media data, RWD contains unstructured data, and recent research seeks to unlock value from such data in addition to the structured data in electronic healthcare records. Natural language processing (NLP) and more sophisticated ML is particularly being applied to knowledge extraction from clinical notes in electronic healthcare records (Weiss et al., 2018).

Signal detection in spontaneous reports (Voss et al., 2017; Peng et al., 2020) and data ingestion (Gurulingappa et al., 2013; Abatemarco et al., 2018; Schmider et al., 2019; Routray et al., 2020) represent just under one-fifth (18%, 6/33) of the industry-associated papers included in the review. This aligns with our expectation that routine PV operations utilize ML to automate and improve capabilities. Literature review, which is a routine PV activity, is represented by approximately 6% (2/33) of the papers (Gurulingappa et al., 2012b; Christensson et al., 2012). The remaining studies (12%, 4/33) focused on individual, disease-specific safety issues (Yang et al., 2009; Ratcliffe et al., 2010; Suzuki et al., 2015; Antonazzo et al., 2018).

When compared with the pharmaceutical industry in general, and PV functions in particular, the use of ML appears to be further advanced in other sectors and/or industries, such as manufacturing, finance, and air transportation (Kenyon, 2021; TrifectaDirectory.com, 2021). Given the complexity of medicine, it is difficult, if not impossible, to capture the relevant information in rules (Schwartz et al., 1987) and, given the complexity of PV (Ghosh et al., 2020; Lewis and McCallum, 2020) coupled with large volumes of data, it seems logical that automation and ML will eventually be used routinely and widely in this sector.

Our own experience, and the results of our analysis of the scoping review by Kompa et al. (2022) show that industry is, at this time, very interested in applying ML to the PV lifecycle. As ML tools improve, we expect that they will demonstrate their value above and beyond traditional software and procedural approaches to PV functions, as is already occurring for routine use of specific tasks, such as employing NLP for more effective screening of literature articles for identifying mentions of suspected adverse events (Glaser et al., 2021). Confidence in, and alignment for the need for such tools is necessary to demonstrate value and engender trust, particularly from regulators. We anticipate that improvements in ML will produce clear benefits to the PV system and may enhance patient safety.

The myriad of overlapping guidance documentation provided to industry causes many to ponder what industry should do, especially if regulatory authorities have different views on the need for and capabilities of ML-enabled PV functions. Clearly fair and safe systems that are trusted by all stakeholders are needed. However, attempting to satisfy all stakeholders may result in a loss of efficiency and the work required to maintain a multifaceted ML-based PV system might exceed the value of the system itself. It is hoped that public-private partnerships such as the Council for International Organizations of Medical Sciences (CIOMS) (Tsintis and La Mache, 2004) may be useful in plotting a course. Indeed, CIOMS recently launched an initiative on AI (Working Group XIV Artificial Intelligence, 2023).

There are many barriers to widespread adoption of ML in PV. These barriers include: the heterogenous nature of PV data; difficulty interpreting the output of ML algorithms; and the lack of performance criteria to determine the acceptability of the output of ML algorithms (Bate and Hobbiger, 2021). Recently, Kassekert et al. (2022) argued that the two major challenges for industry in implementing ML for PV are the risks associated with obtaining adequate training data sets and perceived risk in an emerging regulatory environment (Supplementary Material).

For ML-based systems to succeed in PV, they need to be highly efficient, capable of handling rapid changes in volumes of safety reports and able to learn and incorporate human-in-the-loop mechanisms for identifying novel or unusual patterns and activity. Effective ML should be capable of distinguishing exceptions for human review which may be indicative of data quality issues or a new emerging safety issue (Kjoersvik and Bate, 2022). As this technology evolves, it is important to consider best practices for adoption and validation of these systems, along with consistency of their approach (Huysentruyt et al., 2021).

Case studies describing the application of advanced analytics to perform specific tasks in pharmacovigilance are provided in Table 2.

**TABLE 2:** Case studies describing the application of advanced analytics to perform specific tasks in pharmacovigilance.

Example	Task	Described capability
Identification of a safety outcome through combining NLP extraction of clinical notes with structured data Walker et al. (2016); Weiss et al. (2018)	Computer-assisted elicitation of rules for case definition from experts, using candidate cases drawn from medical records Walker et al. (2016)	The iterative ML-supported technique produced a case definition that had a sensitivity of 92% and a positive predictive value of 79% compared with clinical review alone
	Assess the added contribution of unstructured data extracted from medical text by NLP for detecting acute liver dysfunction (ALD) in patients with inflammatory bowel disease (IBD) Weiss et al. (2018)	Inclusion of NLP terms identified an additional 9% of ALD-onset dates, with consequent earlier recognition in 5% of cases
Social media listening (Twitter and Facebook) for routine post-marketing safety surveillance Powell et al. (2016)	Process 24 months of data to standardize drug names and vernacular symptoms, and to remove duplicates and noise	The methodology effectively transformed social listening data into a usable format for routine post-marketing safety surveillance
Predict the likelihood of a causal association of an observed drug–reaction combination in Individual Case Causality Assessments (ICSR) Cherkas et al. (2022)	Develop a machine learning-based model	Results show that robust probabilistic modeling of ICSR causality is feasible Predictability of the causality assessment of drug–event pairs compared with clinical judgment using global introspection (AUC 0.924; 95% confidence interval [CI] 0.922–0.927). Sensitivity was 0.900 (95% CI 0.896–0.904), and the PPV was 0.778 (95% CI 0.773–0.783)
Signal validation supported by a machine learning (ML)-based pre-validation step to improve process efficiency and consistency Imran et al. (2022)	Cumulative data for six medicinal products [historic signals of disproportionate reporting (SDR) validations and individual case safety reports were used to train and test a ML model	Prediction accuracy of the model ranged from 83% to 86% over 3 months systematic predictions provided valuable information and assisted safety experts in reviewing the SDRs efficiently and consistently
Attitudes of drug safety professionals towards AI in pharmacovigilance Danysz et al. (2019)	Survey of the general sentiments, expectations and readiness for AI	Survey results suggest that pharmacovigilance professionals wish to use their qualifications, skillsets, and experience in work that provides more value for their efforts. Machine learning algorithms have the potential to enhance DS professionals’ decision-making processes and support more efficient and accurate case processing

It is instructive to see how ML is used for safety purposes in other industries. In the air transportation industry, updating accident models has historically been a cumbersome process because of the long period required to review and digest the information contained in long, detailed and highly technical accident reports prepared by safety specialists (Morais et al., 2019). Morais and colleagues developed an ML tool that uses text recognition and text classification, combined with a support vector machine for classifying text according to a predefined taxonomy, to create a ‘virtual risk expert’ that automatically extracts relevant information from accident reports. The Bayesian network tool was trained on several previous accident reports, while the report for the 2018 Lion Air Boeing 737-8 Max accident provided an opportunity to show the feasibility of the tool for rapidly updating an existing accident model. When the ‘virtual risk expert’ was trained exclusively with aviation safety reports it achieved 85% accuracy, whereas if chemical safety reports were included, it achieved 91% accuracy, showing the value of cross-discipline knowledge transfer.

## 3 Discussion

There has been a marked increase in the use of ML to perform PV functions in the pharmaceutical industry, not just for signal detection, but across the PV lifecycle. The supplementary value of ML when combined with rules-based approaches for fields as broad and complex as medicine, and therefore implicitly PV, makes the inevitability of more widespread ML clear (Schwartz et al., 1987; Rajkomar et al., 2019). As ML use in PV matures, we anticipate seeing even more research, of higher quality and with a greater impact that will eventually lead to routine use of this technology.

While the opportunities are clear, challenges remain to more widespread use of ML across the entire PV lifecycle.

In their scoping review, Kompa et al. (2022) highlighted attributes considered to be best practices in the ML literature. These include appropriate inductive biases, no obvious test-train leakage, tuning hyperparameters, and cross validation. In practical terms, this means using a pre-trained model, rather than building a bespoke model ‘from scratch’, using external information or data, and using data or code that is in the public domain. Among the 393 papers analyzed, 42 (approximately 10%) reflected these best practices. Of note, most studies (73%) reported using ‘off-the-shelf’ methods with little to no problem-specific adaptation or domain knowledge. In our analysis, we identified just one industry-associated paper that clearly reflected these modern best practices. It is important to note that no systematic analysis is exhaustive and inevitably some publications will be missed due to the prespecified criteria limiting the search. Furthermore, not all ML used routinely in industry will be published in papers so there may be some omissions in our perspective resulting from that. Also, as our review focused on publications from a list of the top 80 pharmaceutical companies, we acknowledge there may well be publications from companies outside of this list which are therefore not included in our review. Despite this, we would add that we are unaware of any evidence that suggests any differences in routine ML usage, more broadly and unpublished.

There are many reasons why the use of ML for PV does not more frequently meet or exceed best practice criteria. Whether data even exist or are available is a general challenge in PV, considering for example, the situation in low- and middle-income countries. The sheer volume of data to sift through is often a challenge, especially given the heterogeneity of safety data and issues. Limited access to databases, for privacy reasons or other concerns, is another constraint. These challenges contribute to the discordance between what has been done and what needs to be done to realize the potential of ML in PV.

The TransCelerate Intelligent Automation Opportunities (IAO) and Advancing Safety Analytics (ASA) Initiatives are dedicated to evaluating proposed best practices for the application of interrogative methods to safety data sources (TransCelerate_Biopharma_Inc, 2022). The ASA has issued a white paper that surveyed the current state of signal management, provided a simplified framework that comprises three stages (detection, evaluation and action) and identified best practices (Wisniewski et al., 2020). More recently, ASA initiative members evaluated the extent of redundancy among three adverse event reporting databases [EudraVigilance Data Analysis System (EVDAS), FDA Adverse Event Reporting System (FAERS) and WHO-VigiBase] by determining the presence or absence of signals of disproportionate reporting (SDRs) for 100 selected products. There were no significant differences in the number and types of safety signals detectable in the three databases, which suggests that each database on its own could be used for signal detection purposes (Vogel et al., 2020). More recently the group has quantified the extent of ICSR replication in terms of the same report being sent to the same recipient (van Stekelenborg et al., 2023). More innovative and effective ways of sharing information could be envisaged and to ensure maximal impact of ML-enabled PV, fundamental changes in PV would be needed (Bate and Stegmann, 2021).

The pharmaceutical industry is investing in automation to perform PV functions with ML. Industry possesses the process expertise and can help identify the business needs for the use of ML, but it will require high technology companies with deep ML knowledge to provide subject matter expertise. Progress on the technology side has been slow to accommodate PV functions, and it can be difficult at this point to separate marketing messages from tangible, demonstrable benefits with respect to proposed software solutions (Hauben et al., 2007). Thus, even as technology improves, manual processing of ICSRs will be required for the foreseeable future.

In conclusion technology holds increasing potential for automating PV functions in the pharmaceutical industry. ML-enabled systems hold great promise. To date, progress has been uneven but there are successes. As barriers to development and implementation can be reduced or resolved, routine use of ML to perform PV functions is likely.

## Data Availability

The original contributions presented in the study are included in the article/Supplementary Material, further inquiries can be directed to the corresponding author.
