# Corrigendum: An industry perspective on the use of machine learning in drug and vaccine safety

**DOI:** 10.3389/fdsfr.2023.1244115

**Published:** 2023-12-04

**Authors:** Jeffery L. Painter, Raymond Kassekert, Andrew Bate

**Affiliations:** ^1^ GlaxoSmithKline, Global Safety, Durham, NC, United States; ^2^ GlaxoSmithKline, Global Safety, Upper Providence, PA, United States; ^3^ GlaxoSmithKline, Global Safety, Brentford, Middlesex, United Kingdom; ^4^ London School of Hygiene and Tropical Medicine, London, United Kingdom

**Keywords:** pharmacovigilance, machine learning-ML, drug safety, vaccines safety, artificial intelligence

In the published article, there was an error in Figure 1 as published. The figure failed to include the details on the individual node references. The corrected Figure 1 and its caption appear below.

**FIGURE 1:**
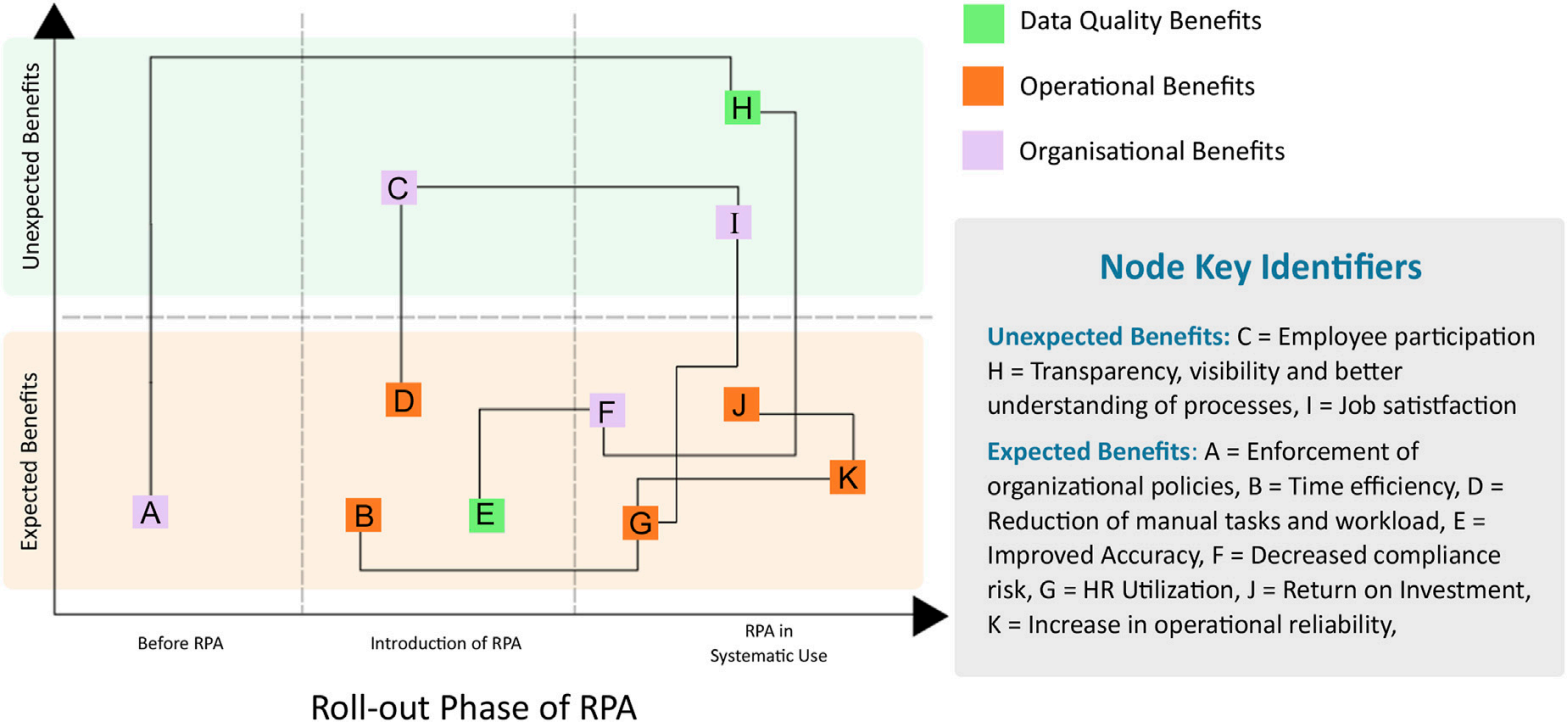
The benefits of robotic process automation (RPA). Additional details are found in Table 1.

In the published article, Table 1 was mistakenly not included in the publication. The missing material appears below.

**TABLE 1:** Details on the benefits of robotic process automation (RPA).

Node	Phase	Benefit	Description
Expected Benefits			
A	Before RPA	Enforcement of organizational policies	Automation defaults to observing organizational policies in a systematic way
B	Introduction of RPA	Improvement in time efficiency	Reduction in time for case processing and booking
D	Introduction of RPA	Reduction of manual tasks and workload	Reduction of repetitive, mundane, tedious manual tasks
E	Introduction of RPA	Improvement in data accuracy	Automate data processing in systematic way. Decrease potential errors and mistakes in safety case review and processing
F	RPA in Systematic Use	Decrease in compliance risk	Timelier fulfillment of compliance and audit requirements for regulatory bodies
G	RPA in Systematic Use	Improvement in human resource utilization	Better utilization of staff, focus on cases which require human intervention
J	RPA in Systematic Use	Return on investment	Increase capability for high volume case processing without increasing staff
K	RPA in Systematic Use	Increase in operational reliability	Allows the organization to operate reliably, even when faced with unexpected challenges
Unexpected Benefits			
C	Introduction of RPA	Improved employee participation	Staff is relieved from repetitive tasks and able to focus on improving overall business processes and decision making
H	RPA in Systematic Use	Improvement in transparency, visibility and better understanding of processes	Business processes and business rules are judiciously executed, processes are documented and explainable to stakeholders
I	RPA in Systematic Use	Improvement in job satisfaction	Empower staff to focus on the most important tasks which lead to better job fulfillment

In the published article, Table 1 should now be updated as **Table 2** and any associated references to Table 1 should refer to **Table 2**, with no changes to its legend or content.

The authors apologize for this errors and state that this does not change the scientific conclusions of the article in any way. The original article has been updated.

