# Supplementary material for: An industry perspective on the use of machine learning in drug and vaccine safety
Source: Front Drug Saf Regul. 2023 Feb 1;3:1110498. doi: 10.3389/fdsfr.2023.1110498 (PMC12443091; doi:10.3389/fdsfr.2023.1110498)
Supplement: Supplementary file 1 [file Table1.docx]

# *Supplementary Material*

Kassekert et al., (Kassekert et al., 2022) identified two specific areas of concern for industry. Firstly, the risks associated with limited training data sets and secondly, the perceived risk in an emerging regulatory environment. In order to investigate the first concern, we examined the sample size included in the 33 industry-associated papers. In order to investigate the second concern we considered the perceived risk to be linked to regulatory expectations. We evaluated the 33 industry-associated papers to determine whether well-established guidance documents were used. Such guidelines at the time of the initial review included: the principles of the International Society for Pharmaceutical Engineering (ISPE) Good Automated Manufacturing Practices (GAMP) (ISPE_GAMP, 2022) and the FDA Good Machine Learning Practices (US_FDA, 2021). We examined the extent of reference to these guidelines as a proxy measure of clarity on expectations of regulatory guidance or well accepted validation.

As shown in the table, the sample size varied widely across these 33 publications (range 844 to 11,100,000). The limited number of studies, applied to different tasks using different ML methods made further inference about the appropriate size of the training set impossible. With respect to Guidance documents, none cited any version of GAMP and none cited any FDA guidance. One study used a social listening platform; the development of the social listening platform had been funded in part by the US FDA under contract with the medical informatics company that had developed the platform, both prior to initiation and continuing throughout the research (Powell et al., 2016). We note that one study reported following standards issued by the American National Standards Institute/American Society for Quality (Routray et al., 2020) These results provide no indication that researchers are aware of, nor following current best practices, although it must be recognised that such practice might be followed implicitly without being referenced.

| **Reference** | **Sample size** | **Guidance documents cited (GAMP or US FDA)** | **Topic Classification** |
| --- | --- | --- | --- |
| (Nordstrom et al., 2007) | 933 | None | Real World Data |
| (Yang et al., 2009) | 7,764 | None | Disease Specific |
| (Ratcliffe et al., 2010) | NA | None | Disease Specific |
| (Gurulingappa et al., 2012b) | 3,000 | None | Literature Review |
| (Christensson et al., 2012) | NA | None | Literature Review |
| (Gurulingappa et al., 2012a) | 2,972 | None | Real World Data |
| (Gurulingappa et al., 2013) | 2,972 | None | Signal Detection |
| (Cao et al., 2013) | 17,433 | None | Real World Data |
| (Cheetham et al., 2014) | 1,239,071 | None | Real World Data |
| (Ferrajolo et al., 2014) | 4,838,146 | None | Real World Data |
| (Yeleswarapu et al., 2014) | 13,500 | None | Real World Data |
| (Jimeno-Yepes et al., 2015) | 1,300 | None | Social Media Data |
| (Suzuki et al., 2015) | 2,275 | None | Disease Specific |
| (Powell et al., 2016) | NA | Development of the social listening platform was funded in part by the US FDA under contract with Epidemico, Inc | Social Media Data |
| (Curtis et al., 2017) | 785,656 | None | Social Media Data |
| (Voss et al., 2017) | 4,480 | None | Signal Detection |
| (Pierce et al., 2017) | 935,246 | None | Social Media Data |
| (Cocos et al., 2017) | 844 | None | Social Media Data |
| (Whalen et al., 2018) | 11,100,000 | None | Real World Data |
| (Antonazzo et al., 2018) | 5,202,124 | None | Disease Specific |
| (Abatemarco et al., 2018) | 20,000 | None | Data Ingestion |
| (Comfort et al., 2018) | 311,189 | None | Social Media Data |
| (Gupta et al., 2018) | 960 | None | Social Media Data |
| (Masino et al., 2018) | 4,402 | None | Social Media Data |
| (Chapman et al., 2019) | 79,004 | None | Real World Data |
| (Gavrielov-Yusim et al., 2019) | 2,500 | None | Social Media Data |
| (Choudhury et al., 2019) | 1,247,722 | None | Real World Data |
| (Schmider et al., 2019) | 100,000 | None | Data Ingestion |
| (Peng et al., 2020) | 1,598 | None | Signal Detection |
| (Wintzell et al., 2020) | 1,310 | None | Real World Data |
| (Routray et al., 2020) | 22,932 | American National Standards Institute/American  Society for Quality. ANSI/ASQ Z1.4-2003 (R2013) | Data Ingestion |
| (Fralick et al., 2021) | 184,865 | None | Real World Data |
| (Gartland et al., 2021) | 15,490 | None | Social Media |

# References

Abatemarco, D., Perera, S., Bao, S.H., Desai, S., Assuncao, B., Tetarenko, N., Danysz, K., Mockute, R., Widdowson, M., Fornarotto, N., Beauchamp, S., Cicirello, S., and Mingle, E. (2018). Training Augmented Intelligent Capabilities for Pharmacovigilance: Applying Deep-learning Approaches to Individual Case Safety Report Processing. *Pharmaceut Med* 32**,** 391-401.

Antonazzo, I.C., Raschi, E., Forcesi, E., Riise, T., Bjornevik, K., Baldin, E., De Ponti, F., and Poluzzi, E. (2018). Multiple sclerosis as an adverse drug reaction: clues from the FDA Adverse Event Reporting System. *Expert Opin Drug Saf* 17**,** 869-874.

Cao, H., Lavange, L.M., Heyse, J.F., Mast, T.C., and Kosorok, M.R. (2013). Medical records-based postmarketing safety evaluation of rare events with uncertain status. *J Biopharm Stat* 23**,** 201-212.

Chapman, A.B., Peterson, K.S., Alba, P.R., Duvall, S.L., and Patterson, O.V. (2019). Detecting Adverse Drug Events with Rapidly Trained Classification Models. *Drug Saf* 42**,** 147-156.

Cheetham, T.C., Lee, J., Hunt, C.M., Niu, F., Reisinger, S., Murray, R., Powell, G., and Papay, J. (2014). An automated causality assessment algorithm to detect drug-induced liver injury in electronic medical record data. *Pharmacoepidemiol Drug Saf* 23**,** 601-608.

Choudhury, O., Park, Y., Salonidis, T., Gkoulalas-Divanis, A., Sylla, I., and Das, A.K. (2019). Predicting Adverse Drug Reactions on Distributed Health Data using Federated Learning. *AMIA Annu Symp Proc* 2019**,** 313-322.

Christensson, C., Gipson, G., Thomas, T., and Weatherall, J. (2012). Text Analytics for Surveillance (TAS):An Interactive Environment for Safety Literature Review. *Drug Information Journal* 46**,** 115-123.

Cocos, A., Fiks, A.G., and Masino, A.J. (2017). Deep learning for pharmacovigilance: recurrent neural network architectures for labeling adverse drug reactions in Twitter posts. *J Am Med Inform Assoc* 24**,** 813-821.

Comfort, S., Perera, S., Hudson, Z., Dorrell, D., Meireis, S., Nagarajan, M., Ramakrishnan, C., and Fine, J. (2018). Sorting Through the Safety Data Haystack: Using Machine Learning to Identify Individual Case Safety Reports in Social-Digital Media. *Drug Saf* 41**,** 579-590.

Curtis, J.R., Chen, L., Higginbotham, P., Nowell, W.B., Gal-Levy, R., Willig, J., Safford, M., Coe, J., O'hara, K., and Sa'adon, R. (2017). Social media for arthritis-related comparative effectiveness and safety research and the impact of direct-to-consumer advertising. *Arthritis Res Ther* 19**,** 48.

Ferrajolo, C., Coloma, P.M., Verhamme, K.M., Schuemie, M.J., De Bie, S., Gini, R., Herings, R., Mazzaglia, G., Picelli, G., Giaquinto, C., Scotti, L., Avillach, P., Pedersen, L., Rossi, F., Capuano, A., Van Der Lei, J., Trifiró, G., and Sturkenboom, M.C. (2014). Signal detection of potentially drug-induced acute liver injury in children using a multi-country healthcare database network. *Drug Saf* 37**,** 99-108.

Fralick, M., Kulldorff, M., Redelmeier, D., Wang, S.V., Vine, S., Schneeweiss, S., and Patorno, E. (2021). A novel data mining application to detect safety signals for newly approved medications in routine care of patients with diabetes. *Endocrinol Diabetes Metab* 4**,** e00237.

Gartland, A., Bate, A., Painter, J.L., Casperson, T.A., and Powell, G.E. (2021). Developing Crowdsourced Training Data Sets for Pharmacovigilance Intelligent Automation. *Drug Saf* 44**,** 373-382.

Gavrielov-Yusim, N., Kürzinger, M.L., Nishikawa, C., Pan, C., Pouget, J., Epstein, L.B., Golant, Y., Tcherny-Lessenot, S., Lin, S., Hamelin, B., and Juhaeri, J. (2019). Comparison of text processing methods in social media-based signal detection. *Pharmacoepidemiol Drug Saf* 28**,** 1309-1317.

Gupta, S., Pawar, S., Ramrakhiyani, N., Palshikar, G.K., and Varma, V. (2018). Semi-Supervised Recurrent Neural Network for Adverse Drug Reaction mention extraction. *BMC Bioinformatics* 19**,** 212.

Gurulingappa, H., Mateen-Rajput, A., and Toldo, L. (2012a). Extraction of potential adverse drug events from medical case reports. *J Biomed Semantics* 3**,** 15.

Gurulingappa, H., Rajput, A.M., Roberts, A., Fluck, J., Hofmann-Apitius, M., and Toldo, L. (2012b). Development of a benchmark corpus to support the automatic extraction of drug-related adverse effects from medical case reports. *J Biomed Inform* 45**,** 885-892.

Gurulingappa, H., Toldo, L., Rajput, A.M., Kors, J.A., Taweel, A., and Tayrouz, Y. (2013). Automatic detection of adverse events to predict drug label changes using text and data mining techniques. *Pharmacoepidemiol Drug Saf* 22**,** 1189-1194.

Ispe_Gamp (2022). GAMP 5 2nd Edition.

Jimeno-Yepes, A., Mackinlay, A., Han, B., and Chen, Q. (2015). Identifying Diseases, Drugs, and Symptoms in Twitter. *Stud Health Technol Inform* 216**,** 643-647.

Kassekert, R., Grabowski, N., Lorenz, D., Schaffer, C., Kempf, D., Roy, P., Kjoersvik, O., Saldana, G., and Elshal, S. (2022). Industry Perspective on Artificial Intelligence/Machine Learning in Pharmacovigilance. *Drug Saf* 45**,** 439-448.

Masino, A.J., Forsyth, D., and Fiks, A.G. (2018). Detecting Adverse Drug Reactions on Twitter with Convolutional Neural Networks and Word Embedding Features. *J Healthc Inform Res* 2**,** 25-43.

Nordstrom, B.L., Norman, H.S., Dube, T.J., Wilcox, M.A., and Walker, A.M. (2007). Identification of abacavir hypersensitivity reaction in health care claims data. *Pharmacoepidemiol Drug Saf* 16**,** 289-296.

Peng, L., Xiao, K., Ottaviani, S., Stebbing, J., and Wang, Y.J. (2020). A real-world disproportionality analysis of FDA Adverse Event Reporting System (FAERS) events for baricitinib. *Expert Opin Drug Saf* 19**,** 1505-1511.

Pierce, C.E., Bouri, K., Pamer, C., Proestel, S., Rodriguez, H.W., Van Le, H., Freifeld, C.C., Brownstein, J.S., Walderhaug, M., Edwards, I.R., and Dasgupta, N. (2017). Evaluation of Facebook and Twitter Monitoring to Detect Safety Signals for Medical Products: An Analysis of Recent FDA Safety Alerts. *Drug Saf* 40**,** 317-331.

Powell, G.E., Seifert, H.A., Reblin, T., Burstein, P.J., Blowers, J., Menius, J.A., Painter, J.L., Thomas, M., Pierce, C.E., Rodriguez, H.W., Brownstein, J.S., Freifeld, C.C., Bell, H.G., and Dasgupta, N. (2016). Social Media Listening for Routine Post-Marketing Safety Surveillance. *Drug Saf* 39**,** 443-454.

Ratcliffe, S., Younus, M., Hauben, M., and Reich, L. (2010). Antidepressants that inhibit neuronal norepinephrine reuptake are not associated with increased spontaneous reporting of cardiomyopathy. *J Psychopharmacol* 24**,** 503-511.

Routray, R., Tetarenko, N., Abu-Assal, C., Mockute, R., Assuncao, B., Chen, H., Bao, S., Danysz, K., Desai, S., Cicirello, S., Willis, V., Alford, S.H., Krishnamurthy, V., and Mingle, E. (2020). Application of Augmented Intelligence for Pharmacovigilance Case Seriousness Determination. *Drug Saf* 43**,** 57-66.

Schmider, J., Kumar, K., Laforest, C., Swankoski, B., Naim, K., and Caubel, P.M. (2019). Innovation in Pharmacovigilance: Use of Artificial Intelligence in Adverse Event Case Processing. *Clin Pharmacol Ther* 105**,** 954-961.

Suzuki, A., Yuen, N.A., Ilic, K., Miller, R.T., Reese, M.J., Brown, H.R., Ambroso, J.I., Falls, J.G., and Hunt, C.M. (2015). Comedications alter drug-induced liver injury reporting frequency: Data mining in the WHO VigiBase™. *Regul Toxicol Pharmacol* 72**,** 481-490.

US_FDA (2021). *US Food and Drug Administration. Good Machine Learning Practice for Medical Device Development: Guiding Principles. October 27* [Online]. Available: <https://www.fda.gov/medical-devices/software-medical-device-samd/good-machine-learning-practice-medical-device-development-guiding-principles> [Accessed November 15 2022].

Voss, E.A., Boyce, R.D., Ryan, P.B., Van Der Lei, J., Rijnbeek, P.R., and Schuemie, M.J. (2017). Accuracy of an automated knowledge base for identifying drug adverse reactions. *J Biomed Inform* 66**,** 72-81.

Whalen, E., Hauben, M., and Bate, A. (2018). Time Series Disturbance Detection for Hypothesis-Free Signal Detection in Longitudinal Observational Databases. *Drug Saf* 41**,** 565-577.

Wintzell, V., Svanström, H., Melbye, M., Ludvigsson, J.F., Pasternak, B., and Kulldorff, M. (2020). Data Mining for Adverse Events of Tumor Necrosis Factor-Alpha Inhibitors in Pediatric Patients: Tree-Based Scan Statistic Analyses of Danish Nationwide Health Data. *Clin Drug Investig* 40**,** 1147-1154.

Yang, X., Brandenburg, N.A., Freeman, J., Salomon, M.L., Zeldis, J.B., Knight, R.D., and Bwire, R. (2009). Venous thromboembolism in myelodysplastic syndrome patients receiving lenalidomide: results from postmarketing surveillance and data mining techniques. *Clin Drug Investig* 29**,** 161-171.

Yeleswarapu, S., Rao, A., Joseph, T., Saipradeep, V.G., and Srinivasan, R. (2014). A pipeline to extract drug-adverse event pairs from multiple data sources. *BMC Med Inform Decis Mak* 14**,** 13.
