# Supplementary material for: An industry perspective on the use of machine learning in drug and vaccine safety
Source: Front Drug Saf Regul. 2023 Feb 1;3:1110498. doi: 10.3389/fdsfr.2023.1110498 (PMC12443091; doi:10.3389/fdsfr.2023.1110498)
Supplement: Supplementary file 2 [file Table2.docx]

Supplementary Table 2 – Details on the benefits of robotic process automation:

| Node | Phase | Benefit | Description |
| --- | --- | --- | --- |
| Expected Benefits | | | |
| A | Introduction of RPA | Improvement in time efficiency | Reduction in time for case processing and booking |
| B | RPA in Systematic Use | Improvement in human resource utilization | Better utilization of staff, focus on cases which require human intervention. |
| C | Introduction of RPA | Reduction of manual tasks and workload | Reduction of repetitive, mundane, tedious manual tasks |
| D | Introduction of RPA | Improvement in accuracy | Automate processing in systematic way. Decrease potential errors and mistakes in case review and data processing. |
| E | RPA in Systematic Use | Decrease in compliance risk | More timely fulfillment of compliance and audit requirements for regulatory bodies |
| F | RPA in Systematic Use | Return on investment | Increase capability for high volume case processing without increasing staff. |
| G | Before RPA | Increase in operational reliability | Allows the organization to operate reliably, even when faced with unexpected challenges. |
|  | | | |
| Unexpected Benefits | | | |
| H | Introduction of RPA | Improvement in job satisfaction | Empower staff to focus on the most important tasks which lead to better job fulfillment. |
| I | RPA in Systematic Use | Enforcement of organizational policies | Automation defaults to observing organizational policies in a systematic way. |
| J | RPA in Systematic Use | Improvement in transparency, visibility and better understanding of processes | Business processes and business rules are judiciously executed, processes are documented and explainable to stakeholders. |
| K | RPA in Systematic Use | Improved employee participation | Staff is relieved from repetitive tasks and able to focus on improving overall business processes and decision making. |
